# Pectoralis Muscle Transposition in Association with the Ravitch Procedure in the Management of Severe Pectus Excavatum

**DOI:** 10.1097/GOX.0000000000002378

**Published:** 2019-09-10

**Authors:** Alessio Baccarani, Beatrice Aramini, Giovanni Della Casa, Federico Banchelli, Roberto D’Amico, Ciro Ruggiero, Marta Starnoni, Antonio Pedone, Alessandro Stefani, Uliano Morandi, Giorgio De Santis

**Affiliations:** From the * Division of Plastic Surgery, Department of Medical and Surgical Sciences for Children and Adults, University Hospital of Modena, Via Largo del Pozzo n. 71-41124 Modena, Italy; † Division of Thoracic Surgery, Department of Medical and Surgical Sciences for Children and Adults, University Hospital of Modena, Via Largo del Pozzo n. 71-41124 Modena, Italy; ‡ Division of Radiology, Department of Diagnostics, Clinical and Public Health Medicine, University Hospital of Modena, Via Largo del Pozzo n. 71-41124 Modena, Italy; Division of Plastic Surgery, Department of Medical and Surgical Science, University of Modena and Reggio Emilia, Modena, Italy; ¶ Division of Plastic Surgery, Department of Medical and Surgical Science, University of Modena and Reggio Emilia, Modena, Italy.; § Center of Statistic, Department of Medical and Surgical Sciences for Children and Adults, University Hospital of Modena, Via Largo del Pozzo n. 71-41124 Modena, Italy.

## Abstract

Pectus excavatum (PE) is the most common congenital chest wall deformity. PE is sometimes associated with cardiorespiratory impairment, but is often associated with psychological distress, especially for patients in their teenage years. Surgical repair of pectus deformities has been shown to improve both physical limitations and psychosocial well-being in children. The most common surgical approaches for PE treatment are the modified Ravitch technique and the minimally invasive Nuss technique. A technical modification of the Ravitch procedure, which includes bilateral mobilization and midline transposition of the pectoralis muscle flap, is presented here.

**Methods:**

From 2010 to 2016, 12 patients were treated by a modified Ravitch procedure with bilateral mobilization and midline transposition of the pectoralis muscle flap for severe PE. Outcomes, morphological results, and complications were analyzed with respect to this new combined surgical approach.

**Results:**

There was a statistically significant difference between pre- and postoperative values (*P* = 0.0025) of the Haller index at the 18-month follow-up, showing a significant morphological improvement for all treated patients. After surgery, no morbidity and mortality were noted. The mean hospital stay was 7 days, and all patients were discharged without major complications.

**Conclusion:**

This technique significantly improved patients’ postoperative morphological outcomes and significantly reduced long-term complications, such as wound dehiscence, skin thinning, and hardware exposure.

## INTRODUCTION

Pectus excavatum (PE) is the most common congenital chest wall deformity, affecting 1 to 8 in 1,000 live births.^1^ Indications for the surgical correction of congenital chest wall deformities include functional/physiological, cosmetic, and psychosocial reasons. Palpitations, exertional dyspnea, fatigue, and chest pain are commonly reported symptoms attributed to pectus deformities.^2^

Many patients report exercise intolerance and increasing limitations in physical activity, which they attribute to their chest deformity. Some patients with PE have been shown to suffer a dynamic restrictive pulmonary process.^3^

Pectus deformities are often associated with body image issues, especially in patients in their teenage years, and these issues can predispose patients to psychological distress. Surgical repair of pectus deformities was shown to improve both physical limitations and psychosocial well-being in children.^3–5^

The most common surgical approaches for PE treatment are the modified Ravitch technique and the minimally invasive Nuss technique.^4–6^

The first technique for PE repair was proposed by Ravitch in 1949 and is an open technique that requires partial resection of the costal cartilage, xiphoid excision, and osteotomy of the sternum.^6^ Multiple modifications to this procedure have been proposed over time, such as the placement of a metal strut to support the sternum, which is removed within 6 months to 1 year. It was not until nearly half a century later that an alternative surgical option was devised and published.^7,8^

The goal of the Ravitch procedure is to remove abnormal rib cartilage while preserving the perichondrium, allowing regrowth of the rib cartilage to the sternum in a more anatomic fashion. Other key elements in the operation include performing a sternal osteotomy to allow redirection of the sternum and stabilization of the sternum with a metal bar, when necessary.

A modification of the established Ravitch procedure, which is applied to treat symmetric as well as asymmetric forms of PE and carinatum, was established. It requires exposure of the sternum and ribs, removal of abnormal cartilage, and fixation of the sternum in a proper anatomical position with 2 metal bars, 1 inserted into the sternum (Kirschner nail) and 1 perpendicularly (Rush wire) fixed between the bilateral corresponding ribs and the xiphoidal process. The metal bars are left in place for at least a year and then are removed with a second operation. Results have shown this technique to be effective in correcting the deformity but at the expense of a quite invasive and long-lasting surgical procedure associated with 7–10 days of hospitalization and resulting in a long scar on the anterior portion of the chest. Physical activity is also severely restricted for several months as the costal cartilage slowly grows back together. Furthermore, this procedure is associated with a 15%–20% complication rate according to different series.^9^ A number of complications that can be classified as immediate and late-stage may affect open sternochondroplasty. These complications include hemothorax and pneumothorax, infection, seroma, hardware dislocation, exposure, and eventually inadequate correction or deformity recurrence.^9^

Early complications may be prevented or solved in most instances (at the expense of a reoperation); however, late complications, such as soft tissue thinning, skin breakdown with hardware exposure, hardware dislocation, and inadequate sternocostal healing, may severely compromise the outcome, posing a relevant clinical challenge (Table 1).

**Table 1.** Complications of Open Sternochondroplasty

Immediate	Late
Hemothorax	Seroma
Pneumothorax	Infection
Seroma	Hardware dislocation
Infection	Hardware exposure
	Bone instability
	Skin breakdown
	Inadequate correction

From this perspective, a new surgical approach has been devised by our multidisciplinary team. The approach consists of a technical modification of the original open sternochondroplasty technique with the inclusion of bilateral mobilization and midline transposition of the 2 pectoralis muscle flaps.

**Fig. 1.**
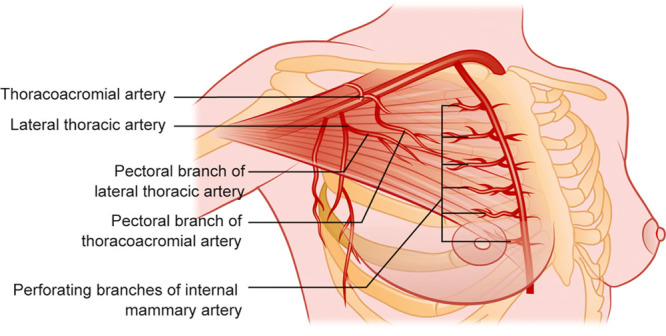
Pectoralis muscle flap and its vascular pedicles.

Pectoralis muscle flap mobilization and transfer is a well-established reconstructive tool for plastic surgeons. It is routinely used in the management of chest defects and for head and neck reconstructions. Flap vascularity is provided by 3 different angiosomes: the thoracoacromial artery, the internal mammary artery, and the lateral thoracic artery (Fig. 1).

Over time, a number of technical modifications of the flap have been described based on different pedicles and have included skin perforator-based components according to the different reconstructive needs.

The authors present a series of 12 patients affected by PE who were surgically treated in our department with open sternochondroplasty associated with bilateral pectoralis muscle transposition and analysis of the outcomes and complications.

## METHODS

This observational retrospective study was approved by the Regional Ethical Committee and the Institutional Review Board of Modena University Hospital. Between 2010 and 2016, 12 patients (2 females and 10 males) who underwent a modified Ravitch procedure for PE treatment in association with bilateral pectoralis muscle transposition were eligible and included in the study. Only patients with preoperative and postoperative CT scans and with at least 1 year of follow-up were included. All patients had already reached the completion of the development of the musculoskeletal system (medium age = 23). The Haller index (HI) (maximal transverse diameter/narrowest AP length of chest) was used to assess the severity of incursion of the sternum into the mediastinum. A normal HI is 2.5. Significant PE has an *index* greater than 3.25, representing the standard for determining candidacy for repair.

Before surgery, all patients underwent blood tests, a troponin value analysis, a CT scan, an ECG, an echocardiogram, and a respiratory function test.

The series was revised to investigate complications, morphological results, and stability of the chest correction.

Morphological results were assessed by comparing preoperative and postoperative HI values from CT scans. The comparison was carried out with a Wilcoxon signed-rank test for paired data. A significance level of *P* < 0.05 was considered.

The final cosmetic outcome was evaluated by 2 independent board-certified plastic surgeons.

A rating scale similar to that published by Humphreys and Jaretzki^10,11^ was used to judge the surgical outcomes. Results were deemed *excellent* when the chest contour was perceived as perfectly normal, with no postoperative sequelae. Results were coded as *good* if the chest contour was comparable to one’s peers but maybe not quite normal and with only minor postoperative sequelae occurring. Results were regarded as *fair* if the chest had partially sunk back. Also termed fair were prominent scars, persistent pain or clicks, or bony “bumps” results were classified as *poor* if the chest appeared as it had preoperatively.^12^

### Surgical Technique

**Fig. 2.**
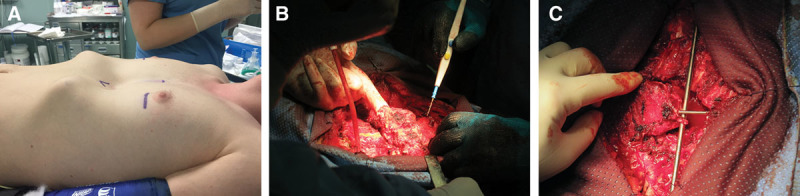
Intraoperative images showing the modified Ravitch procedure. A, Preoperative view. B, Sternum mobilization. C, Hardware insertion.

Under general anesthesia, the patient is placed in a supine position with the hands along the body (Fig. 2A). A Clamshell incision is performed approximately 5 cm below the nipple in males and at the inframammary fold in females. Dissection proceeds to the subcutaneous layer. The fascia is incised at the inferior border of the pectoralis muscles at the level of insertion of the rectus abdominis muscles. The pectoralis muscles are detached inferiorly from the ribs and sternum and are elevated with the skin and subcutaneous plane in 1 layer. The sternum and ribs are thus adequately exposed, taking advantage of the full length of the skin incision. The cartilage is removed from within the perichondrium by using electrocautery and thus resected with care taken to preserve the perichondrium. After the deformed cartilage is removed from the rib to the sternum, the xiphoid process is identified, resected, and elevated, and a blunt digital dissection of the posterior aspect of the sternum is achieved (Fig. 2B). The final sternum mobilization is obtained through a transversal osteotomy of its anterior cortical bone.^6^ This sternal division is usually performed just above the beginning of the sternal depression. Occasionally, 2 sternal osteotomies are required to achieve adequate mobilization. This osteotomy is critical and must be performed carefully in a manner that preserves the continuity of the deep skeletal layer. A subxiphoid space is created, and the sternum is dissected from the underlying pericardium by electrocautery or blunt finger dissection. The intercostal bundles are then disconnected from the sternum and may be ligated or preserved. The sternum is elevated, and an anterior transverse wedge osteotomy is performed at the sternal–manubrial junction. The sternum is then osteotomized and elevated to a normal position. Sternal wires can aid in maintaining this position.

With the abnormal cartilage removed and the osteotomy performed, 2 appropriately sized bars are selected. The sternum is elevated anteriorly, and 1 bar is placed inside the sternum (Kirschner wire), and the other (Rush nail) is sutured or tied to the bilateral rib heads using absorbable sutures, such as PDS or Maxon (Fig. 2C).

**Fig. 3.**
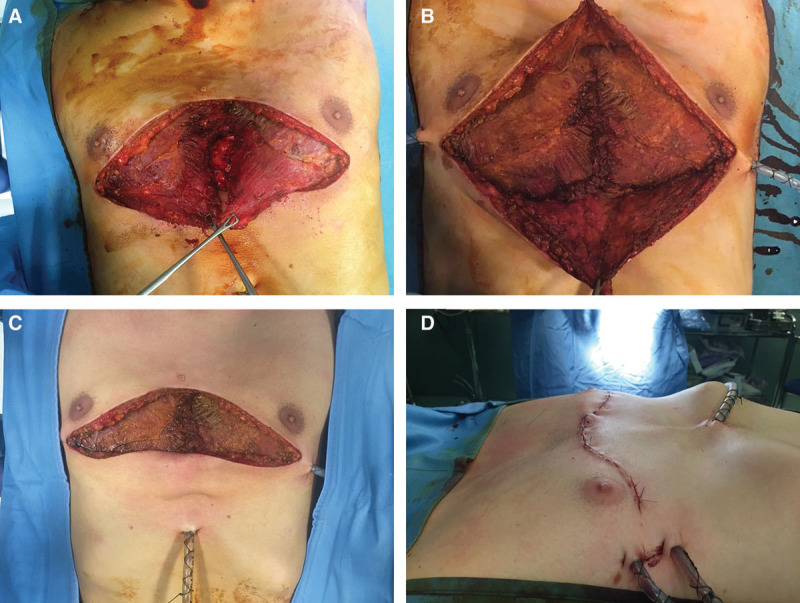
Intraoperative images showing soft tissue muscular coverage. A, Pectoralis muscle flaps are carefully mobilized and detached from the subcutaneous and skin flaps. B, The pectoralis muscles are medially transposed and sutured to one another at the midline and inferiorly attached to the rectus fascia bilaterally to achieve complete coverage of hardware and all osteotomized segments. C, Appearance before skin closure. D, Immediate postoperative view after skin closure showing adequate deformity correction.

After the sternocostal complex has been mobilized, elevated and secured in an appropriate position with hardware, attention is paid to provide soft tissue coverage. Both pectoralis muscles are carefully dissected on a superficial prefascial plane from the overlying skin and subcutaneous layer. This dissection may be performed with traditional electrocoagulation or with the support of ultrasonic cutting and coagulation device.^13^ When proceeding cranially, care should be taken not to devascularize the skin flap. Skin bleeding and refilling is monitored accordingly while proceeding with the cranial dissection. Alternatively, skin perfusion may be intraoperatively assessed with the support of Spy technology (LifeCell Corp., Branchburg, N.J.). Pectoralis muscles are elevated, and the thoracoacromial pedicle is identified and preserved. Muscles are then mobilized as needed to reach a comfortable lateral-to-medial rotation/transposition. To do so, both muscles are divided laterally from the humeral insertion, paying attention not to injure the thoracoacromial pedicle. Once the flaps have been fully mobilized, hemostasis is accurately controlled, and the 2 flaps are sutured to one another medially with PDS sutures (Fig. 3A). With this, full muscular coverage of the osteotomized sternum and ribs is obtained. Hardware is also almost fully protected by this maneuver. Two submuscular drains are inserted, and the muscles are sutured inferiorly to the deep fascia or to the rectus muscle fascia to obtain a complete muscular coverage of all the underlying elements, as shown in Figure 3B, C. Final closure is thus obtained with skin sutures in a double layer (Fig. 3D).

A chest x-ray and blood test were performed after surgery and before discharge from the hospital. Wound dressing was changed every day until discharge. One week after discharge, patients returned to our center for medical assessment and wound evaluation. A chest x-ray was performed after 1 month and 6 months following surgery. The wires were removed under general anesthesia after 1 year with a return to a normal physical activity for the patient. A CT scan was performed at the 18-month follow-up (6 months after hardware removal).

## RESULTS

The average preoperative HI was 6.9 (SD = 4.4), and the average postoperative HI was 4.5 (SD = 1.3). There was a statistically significant difference between pre- and postoperative values (*P* = 0.0025) at the 18-month follow-up. After surgery, no morbidity or mortality were noted. Patients were discharged from the hospital without major complications. The mean hospital stay was 7 days. The results are summarized in Table 2. One patient had a focal dehiscence of the wound with fluid collection during the hospital stay and was immediately debrided and drained. VAC therapy was applied for 4 days, and the wound closed again with no hardware removal. The patient’s discharge was delayed by 3 days. The late postoperative course was uneventful, but there was a residual depression at the 18-month follow-up, and the patient was only partially satisfied with the result.

**Table 2.** Pre- and Postoperative Values of the Haller Index for All Patients

Patients (Pt)	Age	Sex	HI Preoperative	HI Postoperative
Pt 1	20	F	19.6	5.3
Pt 2	20	M	7.5	6.3
Pt 3	37	F	3.8	2.6
Pt 4	23	M	4.7	3.9
Pt 5	22	M	5.5	4.5
Pt 6	18	M	9.8	5.5
Pt 7	22	M	6.1	4.2
Pt 8	19	M	5.5	4
Pt 9	18	M	8.6	7
Pt 10	25	M	3.3	3.2
Pt 11	23	M	4	3.7
Pt 12	29	M	4.8	3.3

HI is defined as the maximal transverse diameter/narrowest AP length of the chest. A normal Haller Index value is approximately 2.5.

Morphological results assessed by 2 independent plastic surgeons revealed good to excellent outcomes in 10 cases, a poor result in 1 case and a fair result in 1 case at the 18-month follow-up.

**Fig. 4.**
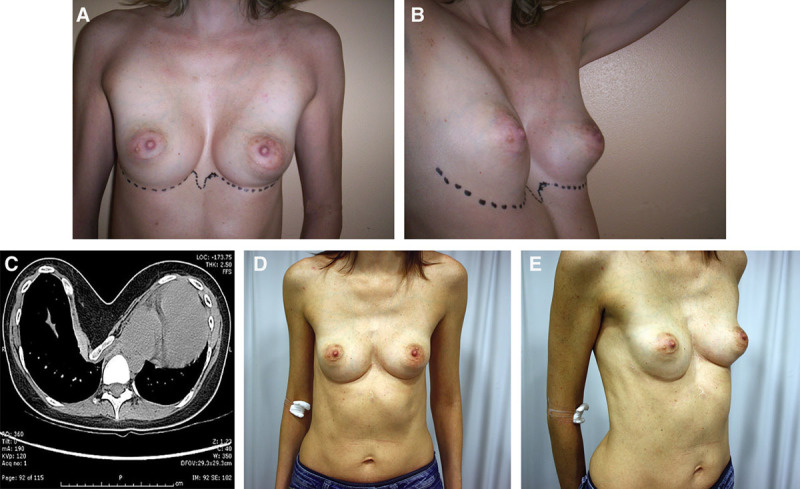
Case 1: A 30-year-old woman showing severe pectus deformity with functional impairment. A and B, Preoperative view of the patient. C, Preoperative CT scan of the chest showing limited anteroposterior diameter. D and E, Postoperative view at 18 months showing adequate and stable correction of the deformity.

**Fig. 5.**
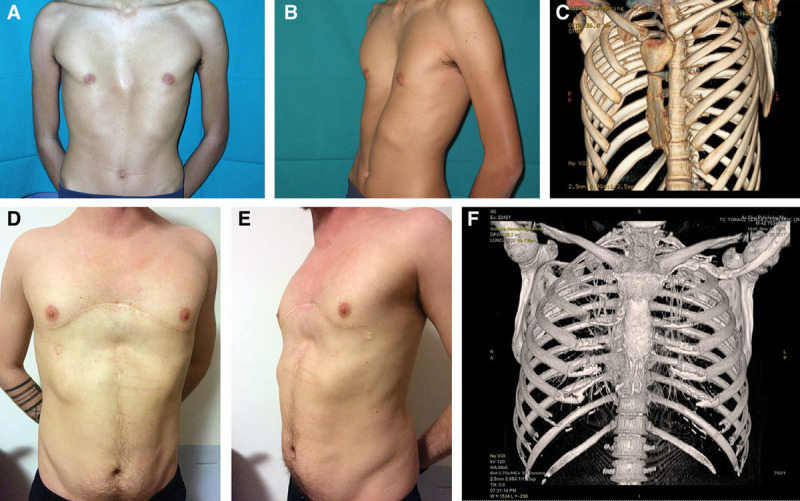
Case 2: A 26-year-old man showing severe pectus deformity with limited functional impairment. A and B, Preoperative view. C, Preoperative 3D CT scan of the chest. D and E, Late postoperative view showing stable correction. F, 18-month follow-up 3D-CT scans after correction.

No skin thinning or breakdown and hardware exposure occurred in this series (Figs. 4, 5).

## DISCUSSION

After the first documented surgical correction of PE by Meyer, Ravitch developed a trend-setting open intervention technique in 1949.^4^ The open approach of subperichondrial resection of all deformed costal cartilage, xiphoid resection, and sternum osteotomy with anterior fixation represented the gold standard through the beginning of the last decade. In 1998, Nuss et al.^5^ introduced a minimally invasive technique as an alternative to standard open repair. The Nuss procedure raises the sternum with a retrosternal metallic bar, which is inserted thoracoscopically and is based on the flexibility of the chest in young patients. Effective correction is possible without the need for extensive costal cartilage resection or sternal osteotomy. Other operational techniques described in the literature for the correction of PE are the method of Leonard or Robicsek, which both represent modifications of the original Ravitch operation.^8^ A number of other minimally invasive approaches have also been described.^5^ Taking the different treatment options together, it has become obvious that not all surgical methods are applicable for all manifestations of PE. In this context, Harrison et al. demonstrated that asymmetry of the sternum poses significant problems for most minimally invasive procedures.^14^ According to Coelho et al., sternochondroplasty is predominant in comparison to the Nuss procedure in the case of asymmetric PE.^15^ In a meta-analysis by Nasr et al., the Ravitch procedure revealed lower rates of reoperation and postoperative hemothorax and pneumothorax than those in the Nuss procedure, with the overall complication rates quite similar.^16^ For this reason, the Ravitch procedure was chosen for treating all severe cases in our center. Nevertheless, complications that warrant operative revision result from displacement of the sternum, gross infection that necessitates incision and drainage, and skin breakdown.^17^ Recurrence has been reported in up to 40% of patients.^17^ The Nuss procedure can be performed for recurrent PE regardless of the technique used for the initial repair; however, the Ravitch procedure is still a useful approach for severe recurrences involving sternocostal junction abnormalities and cartilage regrowth under the sternum.^18^ Complications related to stabilizing metal hardware that must be removed are also significant. Metal devices can shift and migrate into neighboring tissue.^19^ Furthermore, the introduced material can also cause problems such as postoperative chronic pain.^20^ For this reason, the use of absorbable material has been introduced to avoid a second intervention.^20^ The results of different series turned out to be controversial with respect to stability, recurrence, and other complication rates.^20–22^ Nevertheless, by using biodegradable materials, problems such as mechanical instability, a relevant pH shift due to degradation, and a subsequent relevant inflammatory response should be taken into account.

The purpose of combining bilateral pectoralis muscle rotation/transposition with the Ravitch procedure is threefold. First, a well-vascularized soft tissue layer is provided to protect hardware. Second, the well-vascularized muscle supports cartilage and bone healing of all osteotomized segments, and finally, a further soft tissue bulk is provided to improve the final cosmetic outcome. By adding this straightforward surgical step, most late-stage complications are avoided according to our series.

Finally, and less importantly, the presence of a well-vascularized muscle under the skin in the sternal area provides an adequate background for lipofilling if further cosmetic volume enhancement is needed.

## CONCLUSIONS

Our preliminary experience of combining soft tissue coverage with skeletal remodeling in severe PE deformity is encouraging and shows positive results.
